# The impact of the COVID-19 pandemic on the mental health of healthcare professionals and associated factors: A review of literature

**DOI:** 10.1192/j.eurpsy.2021.690

**Published:** 2021-08-13

**Authors:** S. Sellami, H. Mami, M. Kacem, E. Ben Hriz, S. Ben Fraj, R. Bouzid

**Affiliations:** 1 Department Of Psychiatry, University hospital of Nabeul, Nabeul, Tunisia; 2 Department Of Pedopsychiatry, University hospital of Nabeul, Nabeul, Tunisia

**Keywords:** healthcare workers, mental health, coronavirus, factors

## Abstract

**Introduction:**

The COVID-19 pandemic represents a stressful event for humanity. The spread of this disease mainly affects health professionals and interests them closely.

**Objectives:**

Identify the impact of the COVID-19 pandemic on the mental health of healthcare personnels and associated factors exposing them to increased psychological fragility.

**Methods:**

The current article is a narrative review of the existing literature. A search on electronic database like PubMed was undertaken using the search terms “coronavirus mental health healthcare workers”. 20 articles were included in this review.

**Results:**

Studies revealed that health care workers have prensented considerable psychiatric symptoms such as anxiety, depression, PTSD, stress, insomnia, somatization, mental and physical exhaustion, addiction and obssessive compulsive symptoms. The prevalence of these symptoms varies from study to another with almost a more notable prevalence of anxiety and depressive symptoms. The following factors that predispose to developing mental distress were noted: being a frontline health worker, having direct contact with infected patients, working in a city with a high infection rate, female sex, isolation, being a suspected case, stigmatization, change of organization, lack of materials, lack of information, lack of communication, lack of support, fear of contamination or of contaminating loved ones, having an organic pathology.

**Conclusions:**

This notable impact of the pandemic on the mental health of healthcare workers alerts us as colleagues and civil society to the ultimate need for an urgent adequate and up-to-date intervention to alleviate this distress.

